# A qualitative study on nurses’ experiences of mammography

**DOI:** 10.1097/MD.0000000000050690

**Published:** 2026-09-11

**Authors:** Ayşegül Savaşan, Meltem Meriç, Ümran Dal Yilmaz

**Affiliations:** a Department of Nursing, Faculty of Health Sciences, Izmir Tinaztepe University, Izmir, Türkiye; b Faculty of Nursing, Lokman Hekim University, Ankara, Turkey; c Faculty of Nursing, Near East University, Mersin, Turkey.

**Keywords:** mammography, nurses’ experience, qualitative study

## Abstract

Past experiences, emotions, health behaviors, knowledge levels, and health beliefs about mammography can influence participation in future screening. Although nurses possess the necessary knowledge, their compliance with mammography screening schedules is lacking. This qualitative study explored the experiences of nurses who had undergone mammography screening. This study adopted a qualitative phenomenological research design. The study sample comprised 20 nurses who had undergone mammography in the last year, who were identified through purposive sampling, and who agreed to participate in the study. The study data were collected during in-depth interviews using information garnered from data on the sociodemographic characteristics of the participants and a semi-structured interview form comprising 8 questions. The nurses included in the study had a mean age of 51.05 ± 3.51 years, and 10 had postgraduate degrees. Descriptive and interpretive analyses of the gathered data revealed 4 main themes: deciding to have a mammogram, experiences before the mammography procedure, experiences during the mammography procedure, and experiences after the mammography procedure. Nurses experienced feelings of anxiety, fear, and doubt related to a possible cancer diagnosis before, during, and after their mammography procedures, suggesting the need to plan interventions to eliminate fears associated with mammography procedures among nurses. Targeted interventions such as educational workshops and emotional support programs can help nurses overcome their apprehensions and build confidence in the procedure.

## 1. Introduction

Breast cancer is the most prevalent form of cancer and the leading cause of death among women. According to the Global Cancer Statistics 2020 (GLOBOCAN 2020) data published by the International Agency for Research on Cancer (IARC 2020), breast cancer accounts for 47.7% of all reported cancer cases and ranks among the top 5 cancers affecting women in Türkiye.^[1]^ The reported incidence of breast cancer in Türkiye was 24/1,00,000 in 1993 but surged to 50/1,00,000, more than doubling, by 2017.^[2]^

Early diagnosis of breast cancer through screening programs can significantly affect mortality and morbidity rates and play a crucial role in determining appropriate treatments. Mammography continues to be the most effective screening approach and has been shown to reduce mortality in breast cancer.^[3]^ Previous studies assessing mammography rates among women from diverse cultures and educational backgrounds in various parts of Türkiye have yielded disparate results, although most reported low rates (approximately 10%) of mammography among women^.[4]^

Several factors can influence women’s adherence to regular mammography screening schedules. These include past experiences, emotional factors, health behaviors, knowledge levels, and health beliefs related to mammography, all of which impact participation in future screening.^[5,6]^ While informative training can address issues related to the lack of knowledge of the purpose of mammography screening, dealing with women’s feelings and beliefs about the procedure has proven more challenging. For instance, women may harbor fear of learning about the presence of malignant tumors or receiving a cancer diagnosis after mammography, while others may experience embarrassment during the procedure, both of which are significant barriers to compliance. Interventions aimed at understanding the emotions felt by women during mammography screening are crucial if such obstacles are to be overcome.^[7]^

Nurses are a professional group that should be capable of effectively addressing obstacles preventing women from undergoing mammography. Moreover, they are well-equipped to play an active role in women’s health by organizing, implementing, and evaluating the effectiveness of training programs. Such programs focus on informing women about mammography, raising awareness, and promoting the integration of mammography into routine practice. Despite the knowledge level of nurses in this area, studies indicate that nurses themselves may miss regular mammography screening. Despite a sound understanding of the benefits of early breast cancer detection methods, including mammography, studies have reported a low level of mammography compliance among nurses.^[8]^ In a 2020 study by Bakir and Demir, 64.8% of the nursing respondents demonstrated high levels of concern about breast cancer; 94% of those aged 35 years and older who participated in the study had never undergone mammography^.[9]^ In another study, the perceived benefits of mammography among nurses scored 18.79 ± 3.23, while their perception of mammography barriers was measured at 26.81 ± 5.75.^[10]^

Due to the nature of their profession, nurses work closely with patients with breast cancer and frequently play a role in guiding women on preventive measures such as mammography. However, the utilization of early detection services for breast cancer among nurses is not desirable.^[11-13]^

Our study took a qualitative view of the mammography experiences of nurses and the reflection of these experiences on their professional roles. Although there have been many studies on breast cancer screening in the literature, their focus is usually on the general female population and tends not to address the mammography experiences of healthcare professionals such as nurses. In particular, there have been limited in-depth studies on nurses’ compliance with mammography screening and the personal and emotional barriers they experience. This gap in the literature can be considered an important deficiency, suggesting that nurses’ habits related to their own health and capacity to inform and guide patients may be lacking. Among the reasons why nurses avoid mammography procedures are perceptions that they are unnecessary,^[12]^ fear of painful procedures^[13]^ and anxiety regarding breast cancer associated with their professional knowledge.^[9]^ Consequently, we believe that, as both women and members of a profession with a female-guiding mission, it is important to explore the experiences of nurses with mammography, their perceptions of mammography procedures, and the meanings they attribute to these procedures. Exploring nurses’ feelings and perceptions related to this issue could add a new dimension to the understanding of mammography practices and lead to better training and support mechanisms for nurses and patients alike.

The present study seeks to understand nurses’ attitudes and behaviors towards their own mammography procedures by exploring their feelings before, during, and after the procedure, their perceptions, the meanings they attribute to these experiences, and the problems they encounter.

## 2. Materials and methods

### 2.1. Study design

Husserl’s descriptive phenomenological research approach was adopted to reveal the views of nurses on mammography.^[14]^ Husserl’s phenomenological method emphasizes the importance of “bracketing” as a means of suspending preconceived notions, and acknowledges the complexity of humans in seeking to understand their experiences from their own perspectives and how they are actually lived. Phenomenology seeks to define what an experience means to the person who is experiencing it and to come up with a comprehensive description of what they experience.^[15]^ The researchers used a reflective diary to document their thoughts, feelings, and perceptions throughout the research.

### 2.2. Study setting and sample

Nurses employed in a university hospital in the Turkish Republic of Northern Cyprus between January and May 2021 were recruited for the study through a purposive sampling approach. Nurses who had undergone mammography in the last year and agreed to participate in the study understood that they could withdraw at any time without any consequences. This is a fundamental principle of the research approach that was upheld throughout the study, although none of the participants declined to participate, and none dropped out.

### 2.3. Data collection

To minimize potential bias or prejudice during data collection, the researchers reflected on their own experiences and documented the information prior to the participant interviews. This made it possible to separate the personal experiences of the researcher from those of the participants – a method known as “bracketing.” For the study, data were collected through an in-depth interview method using an Information Form to garner details of the sociodemographic characteristics of the respondents and a ssemi-structured interview form comprising 8 questions for the collection of study data (Table 1). The researchers first asked the participants if they had undergone a mammogram in the previous year. Those who replied positively were informed about the study and invited to participate. A qualitative research design was adopted because of the flexibility of the approach in terms of the sample size, which was determined based on the research question and purpose.^[16]^ The study was completed by 20 nurses, and data saturation was reached when no further themes or ideas came to light.

**Table 1:** Semi-structured interview form.

Questions
How did you feel when your doctor informed you that you needed a mammogram?
What is the most important thing motivating you to have a mammogram other than the doctor’s recommendation?
How did you feel before the mammogram, what went through your mind?
What emotions did you experience during the procedure, what were you thinking about?
What did you feel and what did you think about while waiting for the mammogram results?
What do you think is the most difficult part of having a mammogram?
What do you think are the benefits of mammograms?
What would you like to say to other people who are to have a mammogram?

The interviews were conducted face-to-face and in Turkish by an A.S. (a specialist psychiatric nurse) in a quiet environment containing only the respondent and the researcher. A limit of 40 minutes was set for the interviews with respect to the busy schedules of the respondents, although in practice the interviews lasted 30 to 45 minutes. To ensure standardization, A.S. conducted all interviews with the participants. Each interview was audio-recorded on a voice recorder, and the researcher made notes on the respondents’ facial expressions and behaviors. The recordings were transcribed verbatim and then transformed into hard copies. Each respondent was given a transcription of their interview by hand to allow them to review, comment, and validate the data to ensure that the data accurately reflected their views, contributing to the credibility of the study. The COREQ (Consolidated Criteria for Reporting Qualitative Studies) method was used to create and report the qualitative data.^[17]^ A reflective diary, audit trails, and member checking were used to ensure credibility, dependability, and confirmability. To minimize potential bias during data collection, the researchers reflected on their own experiences and documented the information prior to the interviews with the participants. The researchers kept a detailed record of all decisions made during the research process and their justifications, and recorded the information in a research journal that was kept up-to-date throughout the research process.

### 2.4. Data analysis

Colaizzi’s data analysis approach was deemed appropriate for the study, as it uses components of Husserlian phenomenology that prioritize descriptions of the lived experience.^[18]^ The transcribed texts were read separately by the researchers, during which meaningful and related expressions were determined. Subsequently, short codes reflecting the essence of these expressions were created, and subthemes and main themes were determined. After working initially independently, the researchers compared their themes at this stage, and common themes were determined. Direct quotes from the participant interviews were selected to increase the reliability of the research. Participants were presented with the expressions and asked to confirm whether the expressions matched their own experiences. In this way, both the explicit and implicit contents of the texts were revealed, and the data were enriched. New or pertinent data obtained during the validation of the texts by the respondents were incorporated and adapted to attain congruence with the lived experiences of the study participants, adding further credibility to the process. This study draws deeply from the phenomenological framework to develop a composite description of the essence of the experiences of all the participants.

### 2.5. Ethical procedures

Ethical approval (date: 09/08/2021; Decision No 2021/086) for the study was obtained from the Near East University Ethics Committee. The “Voluntary Informed Consent Form” prepared by the researchers was approved by the participants prior to the interview. The participants were informed that the interviews would be audio recorded and assured of confidentiality and anonymity in their contributions, with no references made to the name, sex, or age of the individual respondents.

## 3. Results

The women included in the study had a mean age of 51.05 ± 3.51 years, and 10 held a postgraduate degree. The majority of participants were married, currently employed, and had at least 1 child. Of the participants, 9 had no chronic illness, and 13 had reached menopause. Significant statements were extracted from the interview transcripts of 20 participants and used to formulate meanings. The data analyzed in this study were grouped into 4 main themes and 10 subthemes consisting of main themes (Table 2).

**Table 2:** Themes and subthemes in the analysis.

Themes	Subthemes
Deciding to have a mammogram	Presence of a risk factor
	For control purposes
	Self-motivation
Experiences before the mammography procedure	Worry about negative outcomes
	Privacy anxiety
	Fear of feeling pain
Experiences during the mammography procedure	Discomfort, embarrassment, anxiety
	Pain
Experiences after the mammography procedure	Anxiety of waiting for the outcome - uncertainty
	Fear of bad news, anxiety

### 3.1. Theme 1. Deciding to have a mammogram

Of the respondents, 9 stated that they attended control mammograms because of the presence of risk factors, and 6 said that they had mammograms for control purposes because they thought it was time. The most important motivation for mammography, other than the doctor’s recommendation is the need to look after 1’s health as a mother.

#### 3.1.1. Presence of risk factors

It was determined that individual or family medical history was a determining factor in the decision to undergo mammography.

*“My mother had rectal cancer when she was 37 and breast cancer when she was 42. My sister had breast cancer when she was 28. So when I went for a cancer panel two years later, the doctor told me to have a mammogram.” (P2*)

Other reasons for performing mammography were breast pain and the discovery of masses.

*“At the time, I had a pain in my chest. It was like a palpable lump. That’s why I had it removed.” (P9*)

*“I thought it was age, but I felt a pain in my chest. After the examination, they decided it was a mass. I had a scan, and there was a mass.” (P16*)

#### 3.1.2. For control purposes

Some nurses emphasized the importance of regular screening after a certain age and considered screening to be necessary, especially after the age of 40 years.

*“It was for control purposes, related to age, and it should be taken after the age of 40. Therefore, I had it done.” (P3*)

*“I had it done annually because I was over 40 years old, believing it was necessary to undergo the procedure once a year.” (P4*)

#### 3.1.3. Self-motivation

One respondent stated that she was motivated by her responsibility as a mother and that her controlling personality was a further motivation, whereas another respondent said that they were motivated by their professional experience.

*“The most important factor is that I am a mother. I have more concerns since having a child, and I think it is necessary to be cautious in life. I have a somewhat controlling character. I know I cannot control everything, but I still need to do something about preventable diseases with early diagnosis.” (P4*)

*“I was an angiography nurse, and I also worked in coronary intensive care. You know, when we work in angiography, we are exposed to radiation. Cancer has spread a lot in society, so I can say that it is to protect my own health and take precautions by thinking about my own health.” (P13*)

### 3.2. Theme 2. Experiences before the mammography procedure

A significant number of the respondents said that they were initially apprehensive after their physicians recommended mammography, making such statements as: “I wonder if there is a problem” or “What will be the result?” This leads to intense anxiety. Most women reported feelings of fear, anxiety, and uneasiness before their mammography procedures.

#### 3.2.1. Worry about negative outcomes

Anxiety and fear associated with waiting for mammography results were often mentioned. One respondent stated that her nursing experience contributed to the intense anxiety she felt throughout the process and her concerns about a potentially bad outcome.

*“Of course, I was worried. I wondered if something would come out, if something malignant would be detected. Since my field is surgical nursing, I am familiar with the cases, aware of the entire process that can unfold. I knew what I would potentially go through, and it all flashed before my eyes. The thought of going through this experience made me intensely anxious.” (P7*)

Another respondent reported feeling anxious and frightened, and being aware of the possible results of mammography due to her profession.

*“Anxiety. I felt anxiety, more precisely, fear. I mean, I was afraid because I contemplated what could happen or, being in the profession, I knew the potential outcomes of mammography. When you are in this profession, you think about a lot of things, and the worst-case scenario goes through your mind.” (P9*)

One of the respondent nurses said that she feared for her children and what would happen next in the event of a negative result.

*“What would happen if something like this happened to me? What would I do? Which doctor would I go to? I have children. How does the process work? Is it too early? At what stage is it? The thought of it being something directly malignant was very scary for me.” (PI6*)

#### 3.2.2. Privacy anxiety

Respondents expressed concerns about whether their confidentiality would be maintained during mammography.

*“Initially, one worries about the safety of the environment, of the room. You are looking at an empty room, wondering if there are any openings into it.” (P15*)

*“It’s actually a bit scary because, I mean, it’s a privacy issue. I thought it might be difficult to shoot.” (P I3*)

#### 3.2.3. Fear of feeling pain

Some of the respondents expressed concern about whether the procedure would be physically painful.

*“Because it is an unfamiliar test, and because I have not experienced it before, you are rightly worried, I wonder how it will be, will it hurt? Will anything come out in the end?” (P13*)

*“You get stressed, there are troubles, I wonder if I will experience it, will it hurt when I do it, will there be too much pain? You know it might hurt a little bit, but will there be a problem? You think of all these things at that moment.” (P18*)

### 3.3. Theme 3. Experiences during mammography procedures

Most respondents reported that mammography was uncomfortable. There were also those who reported feelings of intense pain, embarrassment, shame, and anxiety during the mammography.

#### 3.3.1. Discomfort, embarrassment, anxiety

One participant stated that the process was uncomfortable, but that the pain prevented her from feeling embarrassed.

*“I mean, I thought it was difficult, difficult, and uncomfortable. But the pain prevents embarrassment.” (P1*)

Another participant said that she felt embarrassed because her first mammography was conducted by a male health care professional.

*“The first time I had a mammogram, if I remember correctly, I think I was also embarrassed. It was a man who took the mammogram. There was also some embarrassment. But I finished the procedure”.” (P12*)

One of the respondents reported feeling intense anxiety about possible results during and after the mammogram.

*“The procedure itself was uncomfortable for me. And of course, I always wonder what the result will be in my head. Is there something wrong? After the first scan, they said they would look at it with an ultrasound. Then my anxiety increased even more. Oh no, they must have found something. You look at the face of the medical staff. Did he find something? What is his expression like? I had that kind of anxiety, yes.” (P7*)

#### 3.3.2. Pain

The respondents mentioned physical discomfort associated with mammography and the pain they experienced during the scan.

*“Because it is also a bit painful - the compression, the full removal of the armpit, the feeling of being naked - all of that makes it a bit of a thing. It is actually an irritating thing. I felt some pain, especially when they were pulling the armpit side. There is discomfort in being naked. I felt that the most.” (P20*)

*“I couldn’t think of anything other than the pain because it hurt so much. Of course, more than that, you want it to be done quickly at that time. You can’t think of anything else at that moment. I had the feeling that it should be done as soon as possible, and I should get out of here.” (P15*)

Another respondent stated that although she was a nurse, she did not know enough about the procedure, which caused her to feel both anxiety and pain.

*“The hardest part is that I am a nurse, and while I know a lot of things, I wish I had been better informed by the physician or by my nurse friends. I wish I had been informed about how the shooting would take place. I think when a person is sick, regardless of whether they are a nurse or not, they experience the same anxiety and pain.” (P10*)

### 3.4. Theme 4. Experiences after mammography procedure

The respondents reported that the most challenging aspect of waiting for mammography results was the uncertainty and fear of receiving bad news.

#### 3.4.1. Anxiety of waiting for the outcome - uncertainty

The respondents reported that waiting for mammography results was a source of anxiety and worry, with the greatest source of anxiety being the possibility that the result would be negative and that they would receive bad news.

*“We stayed there for about 8-10 women. Most of them left when they were done. They told us to wait. At that time, anxiety was the most intense emotion I felt. You know that studies report that breast cancer can be seen in one in 10 women out of every 8 women. I look at the 10 of us left here. The feeling that I wonder if I am one of those 10 people bothered me a lot. They took the people that remained back inside.” (3*)

*“When the procedure is over, they tell the women to wait outside, we will inform you. The women get a mammogram and start waiting there. Then the technician comes and reads the names. She reads out the names and says these people can go, these people can stay, we will look at them with ultrasound. Being on that list is a very worrisome thing. Staying there makes you feel like, oh my God, they have found something.” (P7*)

*“A pile of women waiting anxiously. We are now waiting in anxiety. I think the longer you wait, the more anxious you get. That time feels so long, maybe a short time is not that long, but it feels long because you are so anxious. Anxiety increases as you wait.” (P15*)

#### 3.4.2. Fear of bad news and anxiety

The respondents reported that the most challenging aspect of waiting for mammography results was the uncertainty and fear of receiving bad news.

*“They did not give the result immediately. In the first days, I was anxious, wondering if something would come out or not. Is it something bad or something good?” (P13*)

*“You have 40 things going through your mind, was it good or bad? Of course, I waited anxiously until the doctor told me, until he did an ultrasound, until he informed me. I waited anxiously.” (P19*)

*“When you wait too long, anxiety increases even more. You are also afraid of something. If you know what is going to happen, you act accordingly. But the uncertainty of what will happen to you makes you more tired and frightened.” (P15*)

## 4. Discussion

The present study investigated the attitudes and behaviors of nurses toward their mammograms, exploring their feelings before, during, and after the procedure, as well as their perceptions, the meanings they attributed to their experiences, and the problems they encountered. The 4 main themes identified were: deciding to have a mammogram, experiences before the mammography procedure, experiences during the mammography procedure, and experiences after the mammography procedure.

### 4.1. Deciding to have a mammogram

The majority of nurses included in the present study stated that they had undergone routine control mammograms due to the presence of risk factors. Nurses with a family history of breast cancer have been reported to be more motivated to undergo screening and have a high level of sensitivity related to breast cancer screening.^[10]^ A study of nurses working in a tertiary healthcare facility found that those who were screened for cancer before the age of 40 years did so due to a family history of breast cancer, a mass in the breast or breast pain, or as a routine control examination.^[19]^ Another study reported that being a woman, genetic predisposition, or the identification of a mass in the breast is the leading cause of risk among nurses who consider themselves at risk of breast cancer.^[20]^

Some of the respondent nurses stated that their mammogram had been a routine control examination because they believed it was time and that they underwent the mammogram after reaching the age of 40. Previous studies have reported higher scores on the Perceived Barriers to Mammography Scale in women aged ≤ 40 years than in those aged ≥ 41 years, indicating a negative correlation between age and barriers to mammography.^[21,22]^ Among the barriers influencing the early detection behaviors of women and reasons for not having a mammogram are lack of information, fear, anxiety, neglect, fear of mass detection, fear of a cancer diagnosis, cost, lack of time, forgetfulness, and embarrassment.^[20,23]^ In the present study, the greatest motivation for mammography was motherhood. Similarly, in an investigation of barriers and facilitators to mammography in Asian women conducted by Momenimovahed et al, attitudes, beliefs, and motherhood were identified as motivators for mammography.^[24]^

### 4.2. Experiences throughout the mammography procedure

The nurses reported feelings of anxiety, fear, and unease regarding a potential cancer diagnosis before, during, and after mammography, as well as significant anxiety and fear, especially after being diagnosed with cancer. Importantly, the nurses’ emotional responses appeared to reflect 2 distinct sources of distress. Procedural concerns were primarily related to anticipated or experienced pain, breast compression, privacy, and uncertainty about how the mammography procedure would be performed. These concerns were generally linked to the immediate mammography experience and were therefore transient and procedure-specific. In contrast, fear of detecting an abnormality or receiving a breast cancer diagnosis represented a deeper and more persistent concern that extended beyond the procedure itself. This distinction was particularly evident during the waiting period for results, when uncertainty about a possible cancer diagnosis became more prominent. Worry and fear of a cancer diagnosis may influence the early detection behaviors of women.^[23]^ Nurses may refrain from having mammograms, as they may perceive them as unnecessary^[12]^ and out of fear of what is known to be a painful procedure.^[13]^ Furthermore, women may delay or avoid examinations like mammography and Pap smears due to the discomfort associated with the procedures.^[25]^ A qualitative study of women over the age of 40 years revealed that fear and anxiety about mammography-related pain and perceived pain were significant barriers to mammography. In the same study, it was reported that women consider mammography procedures uncomfortable, and the negative perceptions gained during previous mammography experiences could be influenced by those earlier negative encounters.^[26]^

While nurses are generally knowledgeable about mammography procedures, it is believed that they should be informed about the process, both because of the change in their role during the procedure and the potential for limited knowledge. In the present study, respondents who were undergoing their first mammogram expressed fear of how the procedure would be conducted, how privacy would be ensured, and whether they would experience any pain. The respondents reported feeling poorly informed or unprepared for the procedure. Limited knowledge of mammography screening procedures can be considered a significant barrier for those with no previous mammography experience. The fear of pain associated with the procedure among women with no screening experience is often worsened by the information provided by women in their social and family circles with prior mammography experience.^[26]^

Nurses undergoing their first mammogram had limited knowledge of the procedure and were not adequately prepared. Midwives and nurses with no previous mammography experience exhibited higher scores on the perceived barriers to mammography scale than those with previous experience. Furthermore, those with no prior mammography experience harbored elevated perceptions of barriers influenced by fears of the unknown and false beliefs about the procedure.^[27]^ Embarrassment has also been identified as a barrier to early detection.^[23]^ In the present study, some respondents voiced their privacy concerns before the procedure. Women may often be reluctant to expose their breasts to health care personnel.^[28]^ The sex and attitudes of mammography staff play a crucial role in the mammography experience and regular screening. Some women prefer female mammography staff owing to privacy concerns.^[26]^ Cultural beliefs and social norms can directly affect women’s access to health services and mammography rates.^[29]^ The low awareness of breast cancer screening and the association of the disease with privacy are among the main factors affecting early diagnosis in Türkiye.^[19,22]^

Most nurses said that the mammography procedure was uncomfortable and that they reported experiencing pain. A significant proportion of women report feeling some level of pain or discomfort during mammography, which is a further barrier to regular mammography.^[30]^ Pain may be related to various factors, including biological factors such as breast tenderness or thickness, expectations of pain, psychological factors such as previous painful mammography experiences and anxiety levels, and staff-related factors such as communication problems, rudeness, and lack of opportunity to ask questions.^[31]^ The discomfort associated with mammography serves as a deterrent factor for screening; women cite pain during mammography as the reason for delaying the procedure and for their reluctance to undergo mammography every year. Even those who have regular mammograms report that they may postpone the procedure because of the associated pain.^[32]^

Breast compression applied during mammography is based on the experience and expertise of the practitioner in achieving optimal image quality and reducing the radiation dose.^[32,33]^ Among mammography practitioners, deciding on the level of compression to be applied is based on a combination of both art and science, where the practitioner’s experience and expertise help define the appropriate balance between compassion and technical excellence. A study of mammography practitioners revealed compassion to be the primary factor in deciding on an appropriate compression force.^[33]^ Cognitive nursing interventions, such as providing information before the procedure, and emotional aspects, such as being on hand to provide women with emotional support during the procedure, have been shown to reduce pain experienced during mammography. Brief nursing interventions are also reported to be effective in reducing anxiety and can aid women in understanding the necessity of breast compression and in considering it a tolerable and temporary procedure.

The statements of the respondent nurse revealed the importance of providing information on when the mammogram results will be available and assessed, when and how the results will be given, how long they will have to wait, and what procedures will be followed if additional tests are needed. Callbacks for additional mammograms or follow-up tests have been reported to increase anxiety and fear related to breast cancer, especially when the test results are delayed.^[34]^ Their health knowledge increases their anxiety regarding breast cancer.^[9]^ This can be attributed to their knowledge of what a positive diagnosis of breast cancer entails and their focus on potentially negative results, leading to increased concerns.

### 4.3. Strength and limitations of the study

The findings of the present study suggest that there is a need to address the fears and anxieties of nurses regarding mammography, both to improve their personal health outcomes and enhance their professional knowledge. By recognizing and mitigating such concerns, healthcare institutions can ensure that their nurses are more comfortable and better informed about the mammography process. This, in turn, can enhance the quality of care they provide to others, including educating women about the need for regular mammographic screening. Targeted interventions, such as educational workshops and emotional support programs, can help nurses overcome their apprehensions and build confidence in the procedure. Thus, they will be better equipped to advocate for and guide their patients through similar experiences, ultimately contributing to improved breast cancer screening rates and early detection outcomes.

Although a small sample size is appropriate for in-depth qualitative research, the sample may not reflect the feelings and thoughts of the entire nursing profession. The small sample size, potential biases in participant selection, the subjective nature of qualitative data, and challenges of generalizing findings to a broader population can thus be considered the main limitations of the present study. The rich and clear perspectives offered by the respondent nurses may be considered sufficiently insightful to encourage the elimination of fears related to mammography among nursing staff.

## 5. Conclusion

The present study revealed that the respondent nurses were compelled to undergo mammograms based on existing risk factors and as part of routine control examinations. They commonly reported a fear of the pain and discomfort associated with mammograms, as well as concerns that something adverse might happen, leading to intense anxiety at every stage of the procedure. Despite their professional knowledge, nurses may require emotional and informational support to overcome these problems. Interventions designed for nurses should also consider the unique challenges associated with shifting from the healthcare professional role to that of a patient during mammography screening. Although professional knowledge may facilitate understanding of the procedure, it may also heighten awareness of potential adverse findings and contribute to anxiety. Therefore, educational and emotional support interventions for nurses should acknowledge their existing clinical knowledge while providing individualized information, opportunities to express concerns without professional expectations, and reassurance regarding privacy and confidentiality. Peer support or counseling provided in a confidential and nonjudgmental environment may further facilitate nurses’ adjustment to the patient role during screening. Positive mammography experiences among nurses could enhance the quality of the guidance provided to patients. Nurses should be knowledgeable about mammography procedures given their profession; however, it is equally important to recognize their need for information and support, irrespective of their nursing status. To increase confidence and reduce such fears, institutions should develop targeted interventions, such as educational workshops and training programs, to raise awareness and address any misconceptions related to mammography. Furthermore, emotional support mechanisms, including peer discussion groups and counseling services, could help nurses manage their anxiety. The clinical environment can also be improved by incorporating relaxation techniques, optimizing patient comfort, and ensuring effective communication during mammography procedures, thus contributing to a more positive experience. Furthermore, organizational support such as workplace health programs and flexible screening opportunities would facilitate regular participation in breast cancer screening. By addressing these concerns and ensuring that nurses receive adequate support, healthcare institutions can improve patient outcomes, enhance participation in screening programs, and promote early detection of breast cancer. Future studies could analyze the effectiveness of these interventions in creating a more supportive and reassuring screening experience for nurses and patients. In addition, considering the privacy concerns experienced by nurses during mammography procedures, developing policies to ensure the protection of privacy and training programs to ensure the sensitivity of mammography personnel may be considered effective approaches to reducing the discomfort experienced by women during such procedures.

## Acknowledgments

We thank the participants of this study for their valuable contribution to this research.

## Author contributions

**Conceptualization:** Ayşegül Savaşan, Meltem Meriç, Ümran Dal Yilmaz.

**Data curation:** Ayşegül Savaşan, Meltem Meriç.

**Formal analysis:** Ayşegül Savaşan, Meltem Meriç.

**Investigation:** Ayşegül Savaşan.

**Methodology:** Meltem Meriç, Ümran Dal Yilmaz.

**Resources:** Ayşegül Savaşan, Meltem Meriç.

**Supervision:** Meltem Meriç.

**Validation:** Ayşegül Savaşan, Meltem Meriç, Ümran Dal Yilmaz.

**Writing – original draft:** Ayşegül Savaşan, Meltem Meriç.

**Writing – review & editing:** Meltem Meriç, Ümran Dal Yilmaz.
